# Spotted! Computer-aided individual photo-identification allows for mark-recapture of invasive spotted lanternfly (*Lycorma delicatula*)

**DOI:** 10.3389/finsc.2023.1112551

**Published:** 2023-02-06

**Authors:** Nadège Belouard, Jocelyn E. Behm

**Affiliations:** ^1^ Integrative Ecology Lab, Center for Biodiversity, Department of Biology, Temple University, Philadelphia, PA, United States; ^2^ ECOBIO (Ecosystèmes, Biodiversité, Evolution), Univ Rennes, CNRS, Rennes, France

**Keywords:** biological invasion, dispersal, individual recognition, movement, pest, photographic mark-recapture, population size

## Abstract

The spotted lanternfly is an invasive pest for which we lack individual movement data due in part to the difficulty posed by individual identification. We developed a computer‐aided method to identify individual adult spotted lanternfly using wing spot patterns from photos processed in the software I3S and demonstrated the method’s accuracy with lab and field validations. Based on 176 individuals in the lab, we showed that digitizing the spots of one wing allowed a 100% reliable individual identification. The errors due to user input and the variation in the angle of the image were largely negligible compared to inter-individual variations. We applied this method in the context of a mark-recapture experiment to assess the feasibility of this method in the field. We initially identified a total of 84 unique spotted lanternflies, 31 of which were recaptured after four hours along with 49 new individuals. We established that the analysis of recaptures can possibly be automated based on scores and may not require systematic visual pairwise comparison. The demonstration of the effectiveness of this method on relatively small sample sizes makes it a promising tool for field experimentation as well as lab manipulations. Once validated on larger datasets and in different contexts, it will provide ample opportunity to collect useful data on spotted lanternfly ecology that can greatly inform management.

## 1 Introduction

The spotted lanternfly (*Lycorma delicatula* (White) (Hemiptera: Fulgoridae), SLF) is an invasive insect in the early stages of its invasion that is spreading across the northeastern United States and has the potential to cause billions of dollars in damage to the wine, timber, and ornamental plant industries due to its phloem-feeding diet (1–3). At the beginning of invasions, population dynamics data is critical for informing management (4) and gaining knowledge on SLF behavior, dispersal capabilities, and demography is one of the central pillars for managing the invasion (1). Much of this information requires tracking the fates of particular individuals, yet we lack an individual identification method to track SLF in a mark-recapture framework. Demonstrating the applicability of photographic mark-recapture on SLF would open a field of research opportunities that would inform the management of this species. In particular, individual movement data would reveal habitat use to inform where control actions should be enacted and the rate of movement can inform how often actions should be enacted as new individuals move into a location (5, 6).

Non-invasive methods are advised for individual identification in wildlife research, not only for ethical reasons, but also because adverse effects associated with handling and marking, such as changes in behavior or survival, may affect mark-recapture estimates (7). Photographic mark-recapture is a cheap and harmless technique that circumvents the drawbacks of physically marking individuals, as it only relies on the inter-individual variability in permanent natural marks that act like “fingerprints” to visually identify individuals. Photographic mark-recapture has been successfully applied to fish (8–10), amphibians (11–13), reptiles (14–16), and also arthropods (17–19). The SLF likely satisfies several required conditions for individual photo-identification (20): adult wings are covered in spots that likely differ in number and position among individuals (**Figure S1**, **Supplementary Material 1**) and the first pair of wings is rigid and unlikely to be distorted in photos. However, whether the inter-individual variability of the wing patterns is sufficient to distinguish individuals must be tested to determine if the technique is suitable for answering scientific questions.

Manual individual photo-identification is a time-consuming technique, since all pairs of photographs must be compared to recognize individuals, a number that increases exponentially with sample size. Several photo-identification programs (e.g. I3S, Wild.ID, APHIS) have been developed to semi-automate this process by allowing users to digitize particular features on images and calculating an index of dissimilarity between features of candidate (unknown) and reference (known) individuals. The software presents users with reference images ranked by similarity for each candidate image, and lets the user decide whether there is a true match. This process considerably facilitates individual photo-identification but still requires a time-consuming step of careful visual comparison from the user to validate correct matches. The original publication for I3S stated that a score of less than 400 indicated a high probability of a possible match (20). To further reduce the amount of user input required, it would be informative to determine if a threshold can be found for scores indicative of non-recaptures, leaving only a fraction of comparisons with intermediate scores to be manually investigated, facilitating the use of this method on highly locally abundant SLF populations.

To use individual photo identification software for non-invasive mark-recapture studies of SLF, three potential obstacles must be overcome. First, biologically, the patterns on the wings must be variable enough among individuals to allow photo-identification itself, even within localities. Indeed, there is no research on whether spot patterns are genetically coded or environmentally driven, which would cause similar spot patterns within localities. Second, technically, when using the software, dissimilarity scores must not be too sensitive to user error in digitizing the wing spots, nor to the positioning of SLF on trees or lightning conditions in the field. Third, logistically, the method must not be too time intensive and require as little user input as possible, which implies that scores themselves should allow for the identification of recaptures and non-recaptures.

We tested whether individual photo-identification is an appropriate method for mark-recapture in SLF. This process involved validation in the lab to assess whether inter-individual variability in wing spot patterns is sufficiently high for individual identification within and among localities and to test the robustness of the method to digitization errors and photos taken at different angles. We complemented the lab validation with a field validation in the form of a test in natural conditions to demonstrate the applicability of this technique to SLF. Finally, we examined the distribution of the pairwise dissimilarity index between recaptures and non-recaptures to further automate the pattern-matching step and reduce the need for user validation.

## 2 Material and methods

### 2.1 Lab validation

#### 2.1.1 Aim

The objective of the lab validation was to test, based on images of labeled SLF, whether there is enough inter-individual variation and minimal digitization error to make individual photo-identification possible in SLF, and automate individual identification using digitization scores. We used the program I3S Classic version 4.0 (9) and partly followed the technique developed by Sacchi and collaborators on lizards (16), and reused for the development of photo-identification on other taxa (11, 17). Specifically, we used the same method of comparing pairwise dissimilarity scores for two digitizations of the same image, digitization of two images of the same individual, and digitization of different individuals, but we modified the statistical analysis of the scores produced (see below).

#### 2.1.2 Images

**Figure 1:**
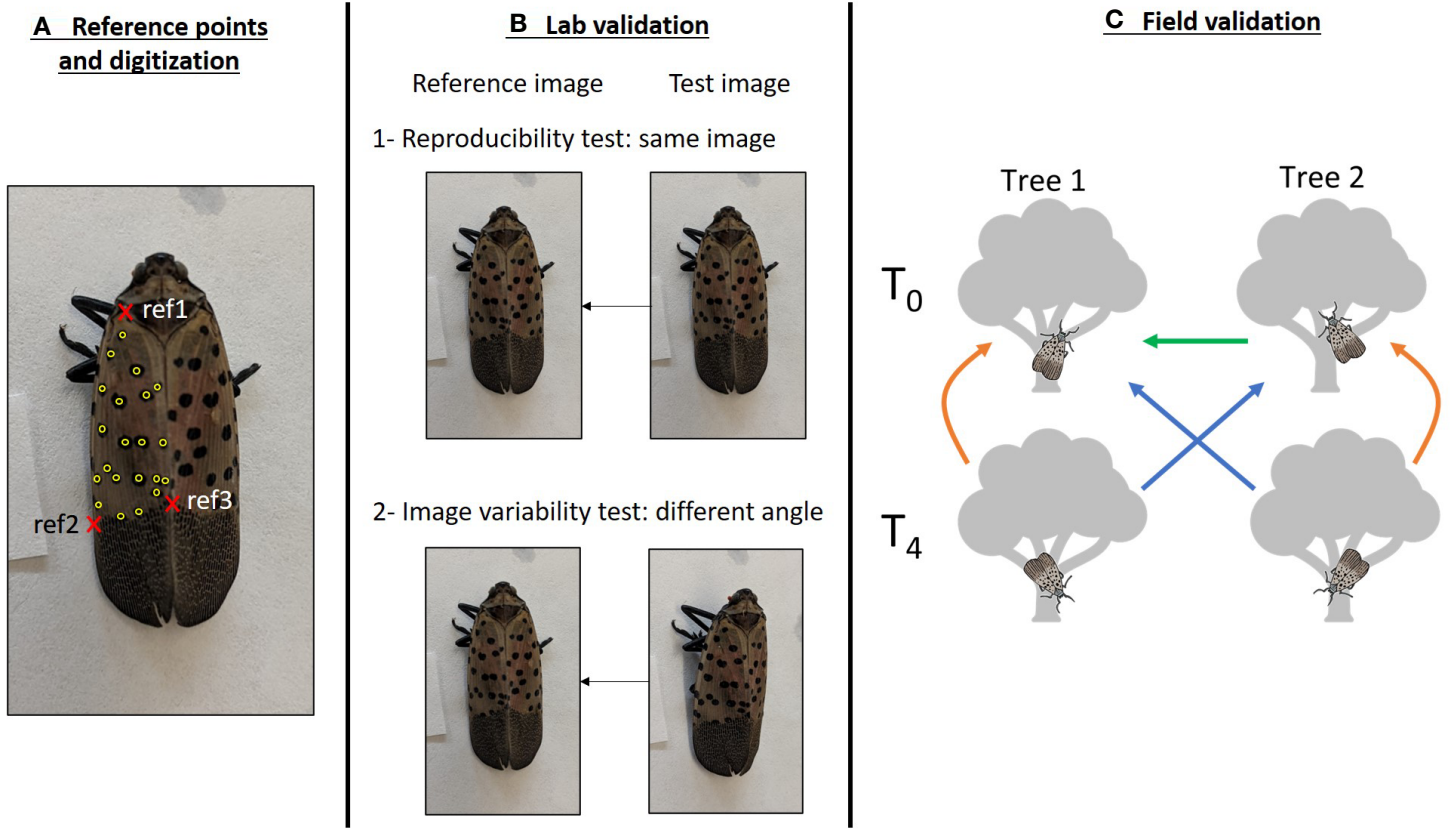
Graphical representation of the methodology used to validate the photo-identification of SLF. **(A)** Detail of the digitization of a SLF individual. The three reference points used to orient the individual are in red: wing attachment point (ref1), left (ref2) and right (ref3) margins of the limit between the spotted and dashed gray zones of the wing. Digitized spots are in yellow. Note that spots on the left margin of the wing were not digitized. **(B)** Lab validation. A first set of photos (top-down set) was digitized twice, the first fingerprint was used as a reference, and the second fingerprint was compared to this reference to assess the reproducibility of the digitization (B1). A second set of photos, taken at an angle (side-angle set), was digitized and compared to the top-down reference to assess the impact of the photo angle (B2). **(C)** Field validation. SLF were photographed on two trees during two sessions (T_0_ and T_4_). Comparisons between images from trees and sessions were performed to assess initial distinctiveness of SLF (T_0 tree1_ vs. T_0 tree2_, green arrow), recaptures on the same tree (orange arrows), and recaptures on a different tree (blue arrows). Arrows point from the candidate set of images to the reference.

We used 176 adult SLF individuals that had been collected in the field at eight locations in Pennsylvania (7-44 per location, **Figure S2**) in 2020 for a companion project. Following capture, individuals were frozen at -20°C and then photographed in the lab with a smartphone. The image resolution was considered sufficient when the contours of the wing(s) considered for recognition as well as the spots were clearly discernible. Two images were taken per individual: the first image was taken from a top-down angle directly above the individual, and the second image was taken at a ~45° angle on the left side of the individual (**Figure 1B**). Individuals are unlikely to be photographed at an angle higher than 45° in the field as the photographer must stay away from the tree to avoid influencing SLF behavior. Therefore, considering the top-down angle and a 45° angle simulates the range of variation in the image angle, and thus the range of image distortion, that may be encountered in the field.

We maintained the top-down and side-angle images of each individual in separate sets to use in the analyses.

#### 2.1.3 Image processing

All images were processed in I3S Classic version 4.0 (20). This program allows the digitization of spot patterns of an animal within an area determined by three reference points that are used to align the images. Together the digitized spot patterns and reference points create a fingerprint file. I3S then compares pairs of fingerprints by superimposing reference points and calculating the distance between pairs of spots. It generates an index of dissimilarity that is the sum of the distance between each spot pair divided by the square of the number of spot pairs (20). As a result, pairs of SLF images with low dissimilarity scores have similar spot patterns.

We limited digitization to the left wing of each individual rather than both wings, given that the number of spots on a single wing typically spans 12-30, a range recommended in I3S to optimize both the identification and the amount of time necessary to digitize images (20). The three reference points used to orient the image were chosen to provide the least distortion of the SLF in the area being digitized: the intersection of the first left rib and the right margin, near the wing attachment point (ref1), and the intersections of the margin and the gray zone at the rear of the wing, on the left (ref2) and right side (ref3, **Figure 1A**). All spots on the left wing were digitized except for the spots on the first left rib that may not be visible depending on the angle of the image as well as on aggregated individuals (**Figure 1A**). While the effect of the number of digitized spots on the accuracy of the identification is an issue that must be addressed in animals with numerous natural marks that are subsampled during digitization (e.g. 16), we did not test this because we digitized all spots within the defined boundary.

The top-down set of images was digitized and used as the reference database. The top-down images were then digitized a second time by another user and matched against the reference database to measure the reproducibility of digitization by two different users using the “batch compare” feature of I3S. “Batch compare” computes scores for all pairwise combinations between the tested images and the reference database. The side-angle set of images was then digitized and matched against the reference database using “batch compare” to measure the method sensitivity to the angle of the image that may distort distances between points.

#### 2.1.4 Statistical analysis

Scores of reference individuals matched to candidate images were called DmatchX, with X representing the rank of the score among all other pairwise comparisons. For example, the reference individual with second-lowest score (i.e., the second most similar image) is called Dmatch2. First, because we knew the correct matches between all pairs of images, we checked the ranking and score of the correct reference image for each candidate image, using the top-down set of images digitized by another user, and then using the side-angle set of images. Most publications consider the method successful if the correct individual is ranked within the first few matches shown by the program (15, 17, 20, 21). Ideally, for the method to be most effective and automated, the reference image corresponding to the candidate image should be ranked first (Dmatch1), so this was our aim for the lab validation. Once we confirmed that Dmatch1 did in fact correspond to the photo of the same individual, we tested whether Dmatch1 was higher for the side-angle set of images than for the top-down set of images due to distance distortion using a Wilcoxon signed rank test.

Second, we assessed how well the software was able to discriminate a specific individual by calculating the difference in scores between Dmatch1 and Dmatch2 (i.e., the correct and the “best” incorrect individual) and comparing it to the difference between Dmatch2 and Dmatch3 (i.e., the two “best” incorrect individuals) for each individual using a Wilcoxon signed rank test. This test indicates if the correct match has a distinctively lower score compared to the best other matches, for each individual. It represents a step further from studies that investigated this question by comparing the first match with the average population score (16, 17, 21), in that the extent of the dissimilarity of the focal individual with the most similar other individual is key in ensuring that the correct individual will always be ranked first. Then, we compared the range of Dmatch1 to that of Dmatch2 to determine whether they do not overlap among individuals, which would allow to set a generic threshold score that indicates recaptures of the same individuals.

Third, we tested whether spot patterns were more similar within localities than among localities, suggesting either heritability or environmentally-driven spot patterns, and making it potentially harder to photo-identify individuals within localities, by comparing Dmatch2 obtained within the locality to Dmatch2 obtained from all localities using Wilcoxon signed rank tests. All statistical analyses were done in R version 4.0.5 (22).

### 2.2 Field validation

#### 2.2.1 Aim

The objective of the field validation was to assess whether this method of photo-identification is robust to the variability in lighting and body positioning of SLF introduced by field conditions and to confirm that the method can be used for photographic mark-recapture of individuals over time.

#### 2.2.2 Images

We photographed SLF found on two red maples (*Acer rubrum*) separated by 12 meters in an urban park in Philadelphia, PA (**Figure S2**) on August 8, 2022 at 3:30 PM (T_0_). SLF visible on tree trunks and lower branches were successively photographed with a smartphone until all adults were photographed, or within a maximum of 5 minutes where abundances were high to standardize sampling effort. During photographing, the photographer avoided getting close enough to the tree to alter the behavior of SLF individuals and zoomed in on the SLF to take photos. Four hours later, the photographer took a second set of photographs on each tree, following the same methods (T_4_, **Figure 1C**). The four sets of images we used in our analyses were tree 1 at T_0_, tree 2 at T_0_, tree 1 at T_4_ and tree 2 at T_4_. Since capture of the same SLF individual in multiple photos within a single photo session was likely, the ability to detect duplicate individuals in a single photo session is an additional piece of information to gauge the accuracy of the software.

#### 2.2.3 Image processing

Because SLF often aggregate on trees, many of the photos were of multiple individuals. Therefore, to facilitate digitization, all images were first cropped to be just of single individuals. All images were then digitized by multiple users using the same three reference points as in the lab validation. We looked for individuals photographed in duplicate during a single session on a tree by visually comparing all pairs of images within a set.

The “batch compare” feature was used to compare sets of images and generate scores. The test of the detection of duplicates within a single session on the same tree was done by matching a set of digitized images against itself. After this step, we kept a single image per individual to create a set of unique individuals with a single image for each session per tree to avoid adding an effect of the number of images per individual. We then assessed the ability to detect recaptures by matching T_4_ images (candidate) to T_0_ images of the same tree and the other tree (reference, **Figure 1C**), as well as matching fingerprints from Tree 2 at T_0_ (candidate) against Tree 1 at T_0_ (reference). In the I3S software, identification of recaptures involves a judgment call based on a visual comparison of all pairs of images, and it is how we determined recaptures, independently of their scores.

#### 2.2.4 Statistical analysis

We first assessed whether visually identified recaptures were classified as Dmatch1 for the corresponding individual. Ideally, if this method could be used in a semi-automated fashion based on scores to identify recaptures, recaptured individuals should have lower Dmatch1 scores and greater differences between Dmatch1 and Dmatch2 than individuals that were not recaptured. To test this, we tested whether Dmatch1 was lower for recaptured than for non-recaptured individuals with a Mann-Whitney test. In addition, we determined whether there was a significant difference between Dmatch1 and Dmatch2 for recaptured individuals compared to non-recaptured individuals with a Mann-Whitney test.

We used the same reasoning to assess whether the program could identify duplicated images within a single session: as Dmatch1 is the same image (score = 0), we checked whether Dmatch2 was the duplicated image, and tested whether Dmatch2 was lower in duplicated images compared to non-duplicated images with a Mann-Whitney test. We determined whether there was a significantly higher difference between Dmatch2 and Dmatch3 for duplicated images than for non-duplicated images with a Mann-Whitney test.

## 3 Results

### 3.1 Lab validation

**Figure 2:**
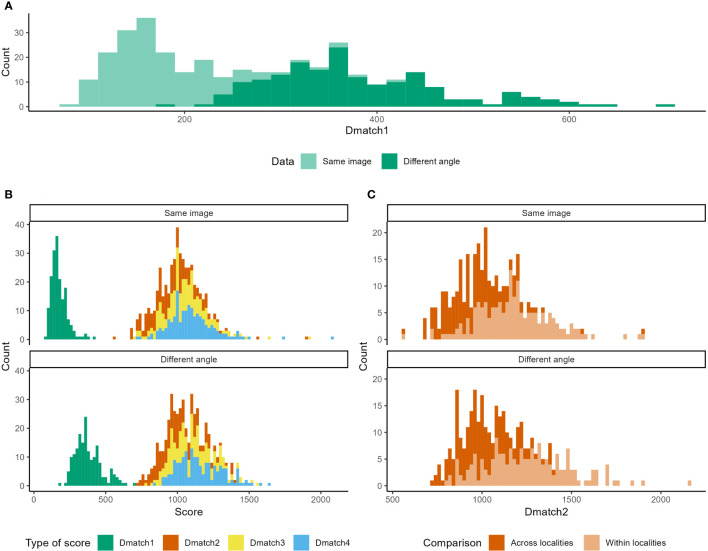
Lab validation of the photo-identification of the spotted lanternfly. **(A)** Distribution of scores of the correct match (Dmatch1) when using the same image or an image taken from a different angle. **(B)** Distribution of scores of the correct (Dmatch1), second-, third- or fourth-best match obtained (Dmatch2, Dmatch3 and Dmatch4, respectively). **(C)** Distribution of scores of the second-best match (Dmatch2) when images are compared to individuals from their locality of origin (within locality) or from all localities combined (across localities).

For the first analysis where we compared the two top-down images digitized by different users, 100% of Dmatch1 for the test images were the correct reference individual among the 176 individuals forming the database. These Dmatch1 scores were higher than 0 (average ± SD: Dmatch1_top-down_ = 176 ± 57), and represented the user error. When we compared side-angle images to the top-down images, 100% of the test images were again matched to the correct reference individual using the program, even if Dmatch1 scores were slightly higher for the side-angle photo set comparison (Dmatch1_side-angle_ = 379 ± 93, V = 0, p < 0.001, **Figure 2A**).

There was an average difference of 783 ± 155 between Dmatch1 and Dmatch2 when comparing the two top-down fingerprints, and 629 ± 161 when comparing the side-angle and top-down fingerprints (**Figure 2B**). These differences were much larger than the differences between Dmatch2 and Dmatch3, which were on average 85 ± 75 for the two top-down fingerprints (V = 15576, p < 0.001) and 89 ± 72 for the side-angle and top-down fingerprints (V = 15575, p < 0.001, **Figure 2B**).

No overlap was found between Dmatch1 and Dmatch2 among all individuals. Dmatch1 scores were always lower than 428 and Dmatch2 scores were larger than 566 with the two top-down images. Dmatch1 were always lower than 705 and Dmatch2 were larger than 719 with the side-angle and top-down images (**Figure 2B**).

When comparing the two top-down images, Dmatch2 scores were lower across localities (Dmatch2_across_ = 960 ± 167) than within localities (Dmatch2_within_ = 1176 ± 208, V = 0, p < 0.001, **Figure 2C**). It was also the case when comparing top-down to side-angle images across versus within localities (Dmatch2_across_ = 1008 ± 147, Dmatch2_within_ = 1247 ± 238, V = 0, p < 0.001, **Figure 2C**).

### 3.2 Field validation

**Figure 3:**
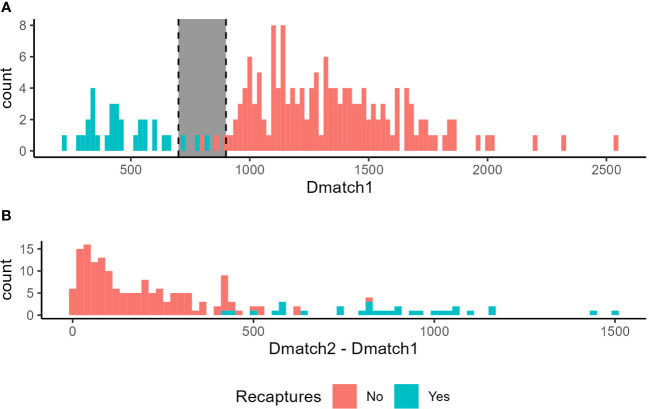
Distribution of scores in the field validation depending on whether the individual is a recapture (blue) or not (red). Note that non-recaptures should have no matching images, so Dmatch1 represents a match with a different individual in that case. **(A)** Scores of the best match (Dmatch1) obtained by individuals. The gray zone corresponds to intermediate scores where Dmatch1 potentially overlaps between recaptures and non-recaptures. **(B)** Difference between the best match (Dmatch1) and the second-best match (Dmatch2).

For Tree 1, 63 and 53 photos of 58 and 45 individuals were taken at T_0_ and T_4_, respectively. For Tree 2, 27 and 44 photos of 26 and 35 individuals were taken at T_0_ and T_4_, respectively (**Table 1**). Based on the visual comparison to identify recaptures: (1) no individual was captured on both trees at T_0_, (2) 34% and 42% of the individuals were recaptured on the same tree at T_4_, and (3) no individual was found on a different tree at T_4_ from the tree it was on at T_0_ (**Table 1**). Recaptured individuals that were visually identified were always ranked as Dmatch1 by the program, meaning that the user would not have to look beyond Dmatch1 to identify recaptures. In the case of recaptures, Dmatch2 was on average 867 ± 262 points higher than Dmatch1 (**Figure 3B**). Dmatch1 and Dmatch2 scores were much closer in the case of non-recaptures (W = 39, p < 0.001) with an average difference of 176 ± 153.

**Table 1:** Summary of the recapture field study.

Tested Reference	Tree 1	Tree 2	
	T_4_ (45 ind.)	T_0_	T_4_ (35 ind.)
Tree 1 T_0_ (58 ind.)	20 (34%) – 828/933	0 (no movement)	0 (no movement)
Tree 2 T_0_ (26 ind.)	0 (no movement)		11 (42%) – 724/807

Number of recaptures between trees and sessions (percentage of initial captures) - Maximal score of recaptures/Minimal score of non-recaptures.

Overall, Dmatch1 showed a clear bimodal distribution, where the first mode consisted of all recaptured individuals, and the second mode was all non-recaptured individuals (**Figure 3A**). In other words, Dmatch1 scores were lower for recaptured individuals than for non-recaptured individuals (Dmatch1_recaptures_ = 473 ± 150, Dmatch1_non-recaptures_ = 1342 ± 300, W = 4742, p < 0.001). There was however a “gray zone” where recapture and non-recapture Dmatch1 scores overlapped, since Dmatch1 of recaptures were lower than 828 and Dmatch1 of non-recaptures were larger than 807 (**Figure 3A**).

For the test of duplicates within a single session, Dmatch2 was always the duplicated individual when it had been identified manually, meaning that duplicated individuals were ranked better than non-duplicated individuals for each individual. In the case of duplicates, Dmatch2 was on average 471 ± 211, significantly lower than the Dmatch2 of non-duplicated individuals (1292 ± 288, W = 6367, p < 0.001, **Figure S3A**). In other words, Dmatch2 showed a bimodal distribution, the first mode consisted of duplicated individuals, and the second mode was all non-duplicated individuals. Dmatch2 and Dmatch3 were much closer in the case of non-duplicated individuals with an average difference of 159 ± 139, while in the case of duplicated individuals this difference was 818 ± 296 (W = 159, p < 0.001, **Figure S3B**). There was a gray zone where duplicated and non-duplicated Dmatch2 overlapped, since Dmatch2 of duplicates were lower than 969 and Dmatch2 of non-duplicates were larger than 741 (**Figure S3A**).

A sensitivity analysis was conducted to assess the impact of sample size on the evolution of the scores of the best-ranked different individuals (**Supplementary Material 2**). It was done by resampling the pool of all individuals (lab and field validation, N = 309) and matching it against itself. The procedure was repeated 10 times, and with different sample sizes (n = 10 to 309). Results show that the lowest score for an incorrect match does not decrease linearly but seems to stabilize around 1000 as sample sizes increase (**Supplementary Material 2**). In all cases, no more than 30% of these incorrect matches had scores < 900 and would have to be visually verified.

## 4 Discussion

Research on the spread of SLF would benefit from having an individual-based identification method to allow for the tracking of individuals in the field and determine demographic parameters of wild populations. Our results show that the wing spot patterns were unique among the tested individuals and that their semi-automated comparison was a reliable method for individual identification. This study thus constitutes a promising proof-of-concept for photographic mark-recapture in the spotted lanternfly.

The errors due to user input and the variation in the angle of the image were largely negligible compared to inter-individual variation. The dissimilarity score for different individuals was high, and lower across localities than within localities (likely because of higher sample sizes), suggesting that inter-individual variability is high enough within localities for the software, potentially making this methodology applicable at both small and large spatial scales. Future work is needed to confirm these patterns across a wider array of localities.

In the case of recaptures in the field, the best match was always the correct individual from our reference database of 84 individuals. This high performance of the method for identifying recaptures is not unique to SLF (8) but seems to exceed what has been found in other species, where correct individuals could be found in the first few best matches of reference databases of 132-358 individuals (10, 16, 17). Moreover, in our dataset, scores of the best match were much lower for recaptures than for non-recaptures, as well as for duplicated images compared to non-duplicated images, suggesting that further automatization of the image analysis is possible to reduce the need for user visual validation. Indeed, the bimodal distribution of the best-match score across individuals suggests an almost clear-cut transition between scores of recaptures and scores of non-recaptures, and between scores of duplicated and non-duplicated images. The original publication for the I3S software stated that a score of less than 400 indicated a high probability of a possible match (20). Based on our analysis, we would expand this to a higher score, and any individual with a best-match score lower than 700 could be considered as a recapture, while an image with a best-match score larger than 900 is likely an unknown individual (non-recapture). Best-match scores in between these values were in a gray zone where recaptures and non-recaptures overlapped. Comparable thresholds were found for duplicated and non-duplicated images (700-1000). Only images with best-match scores in this gray zone would need to be manually checked, potentially drastically reducing the user input time in the analysis to a fraction of the individuals, 3% of the individuals in our field study, compared to the routine use of the program that involves visually checking at least the first match of 100% of the individuals tested.

The limits of this gray zone would have to be calculated and reported on larger sample sizes, in different environmental contexts and times of the day to determine their empirical stability. This method could be less effective for high sample sizes because the more individuals are compared, the more likely it is to find similar individuals. However, our sensitivity analysis suggested that interindividual scores stabilize as sample sizes increase, which supports the conclusions established here on smaller sample sizes (**Supplementary Material 2**). Overall, absolute threshold values for the gray zone are not necessary, but this concept makes processing the data much easier, and researchers applying this method in the future should look for thresholds, which could be study-specific and determined using appropriate pilot studies.

By using the photo identification of individuals, new opportunities abound for research on the SLF, including the study of ecological or biological questions that cannot be answered without individual identification, like the estimation of movements over long periods of time. For example, knowledge on SLF flight or movement capabilities in the field so far have been limited to observations of groups of individuals (23–25). Studying individual movements can provide better estimates on SLF habitat use and dispersal capabilities. Although the field validation component of our study was not intended to produce biological information, we discovered moderately high recapture rates, though no individuals moved between trees despite trees being only 12 m from each other. This may suggest that SLF had limited movements during that day. Expanding this study to compare different host trees and periods of time would bring significant insight into key elements of their ecology which could be used to parameterize first principles mathematical models.

We also demonstrated that the analysis time can be greatly reduced by analyzing scores, leaving only a fraction of photos with intermediate scores for visual validation. We suggest that future projects conduct a pilot study on a subset of individuals to confirm or adapt the thresholds proposed in this article to fit their study case. User input is still needed for digitizing the sets of images, but as current artificial intelligence programs already offer the possibility for automated species identification (26), we believe that this obstacle will soon be overcome, and allow to fully automate digitization, making the photographic mark-recapture process almost immediate. Finally, similar approaches could be adapted and applied to other species that have visual patterns with high interindividual variability.

## Data availability statement

The original contributions presented in the study are included in the article/**Supplementary Material**. Further inquiries can be directed to the corresponding author.

## Author contributions

NB conceptualized the idea, collected the data and digitized the images, performed the analysis and wrote the original draft of the paper. JB helped with digitization of the images and critically reviewed and revised the manuscript. All authors contributed to the article and approved the submitted version.

## Supplementary material

Supplementary Data Sheet 1 Supplementary figures S1, S2 and S3

Supplementary Data Sheet 2 Sensitivity analysis

Supplementary Data Sheet 3 Lab validation data

Supplementary Data Sheet 4 Lab validation data (within populations only)

Supplementary Data Sheet 5 Field validation data
