# Supplementary material for: Spotted! Computer-aided individual photo-identification allows for mark-recapture of invasive spotted lanternfly (Lycorma delicatula)
Source: Front Insect Sci. 2023 Feb 6;3:1112551. doi: 10.3389/finsc.2023.1112551 (PMC10926401; doi:10.3389/finsc.2023.1112551)
Supplement: Supplementary Data Sheet 1 — Supplementary figures S1, S2 and S3 [file DataSheet_1.pdf]

Supplementary Material 1 for manuscript: Spotted! Computer-aided individual photo-identification allows for mark-recapture of invasive spotted lanternfly (*Lycorma delicatula*)

N. Belouard[1,2]*, J.E. Behm[1]

[1]Integrative Ecology Lab, Center for Biodiversity, Department of Biology, Temple University, Philadelphia, Pennsylvania, USA

[2]ECOBIO (Ecosystèmes, Biodiversité, Evolution), Univ Rennes, CNRS, Rennes, France

*** Correspondence:**
Nadège Belouard
nadege.belouard@gmail.com

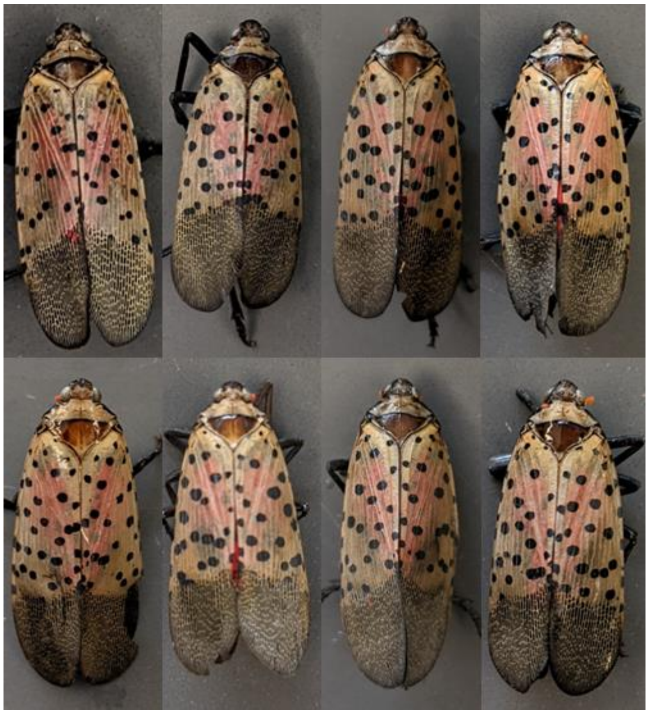

Figure S1. Illustration of the variability in wing spot patterns found in the spotted lanternfly

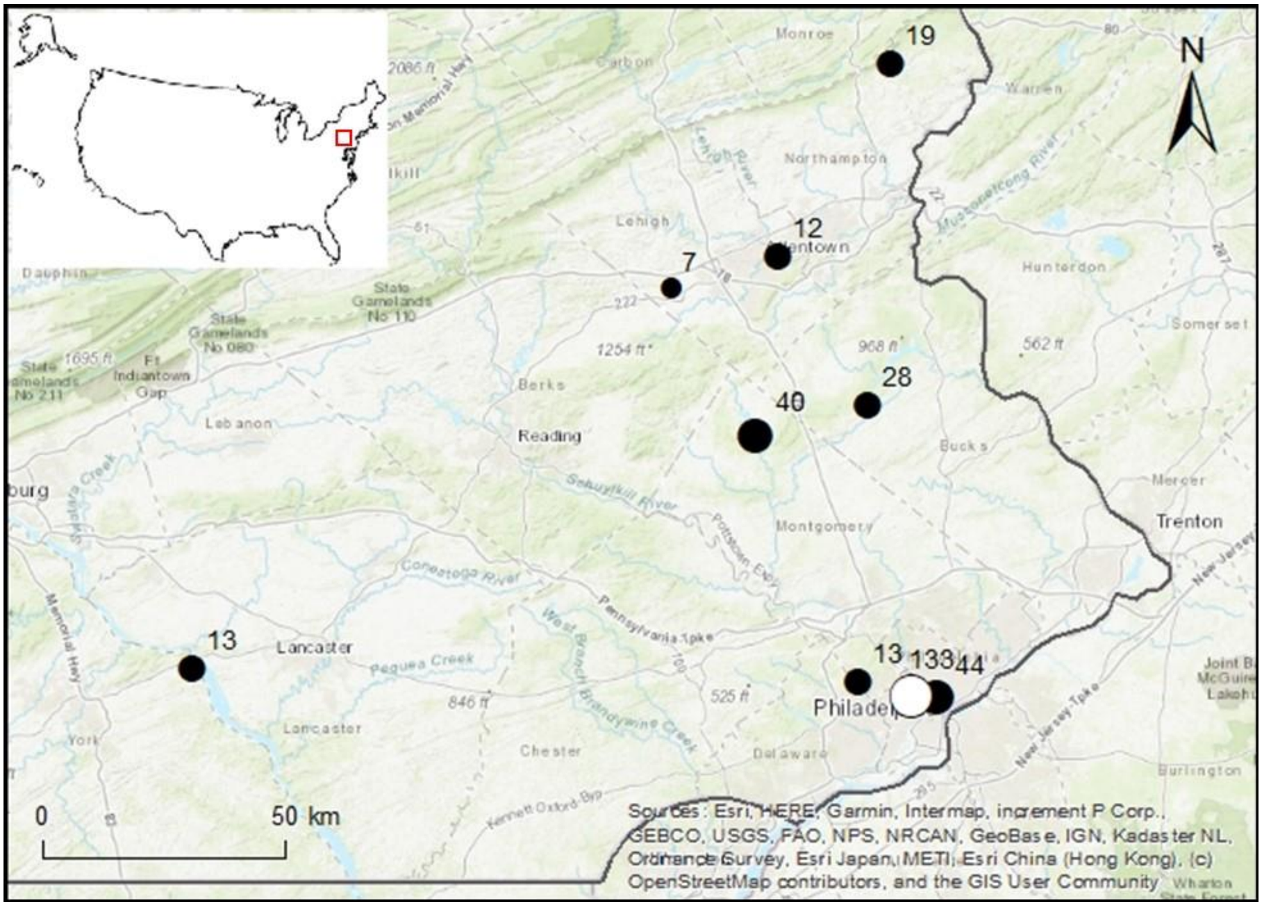

Figure S2. Sampling locations for the lab validation (black circles) and the field validation (white circle) of the photo-identification of the spotted lanternfly in Pennsylvania. Labels are sample sizes.

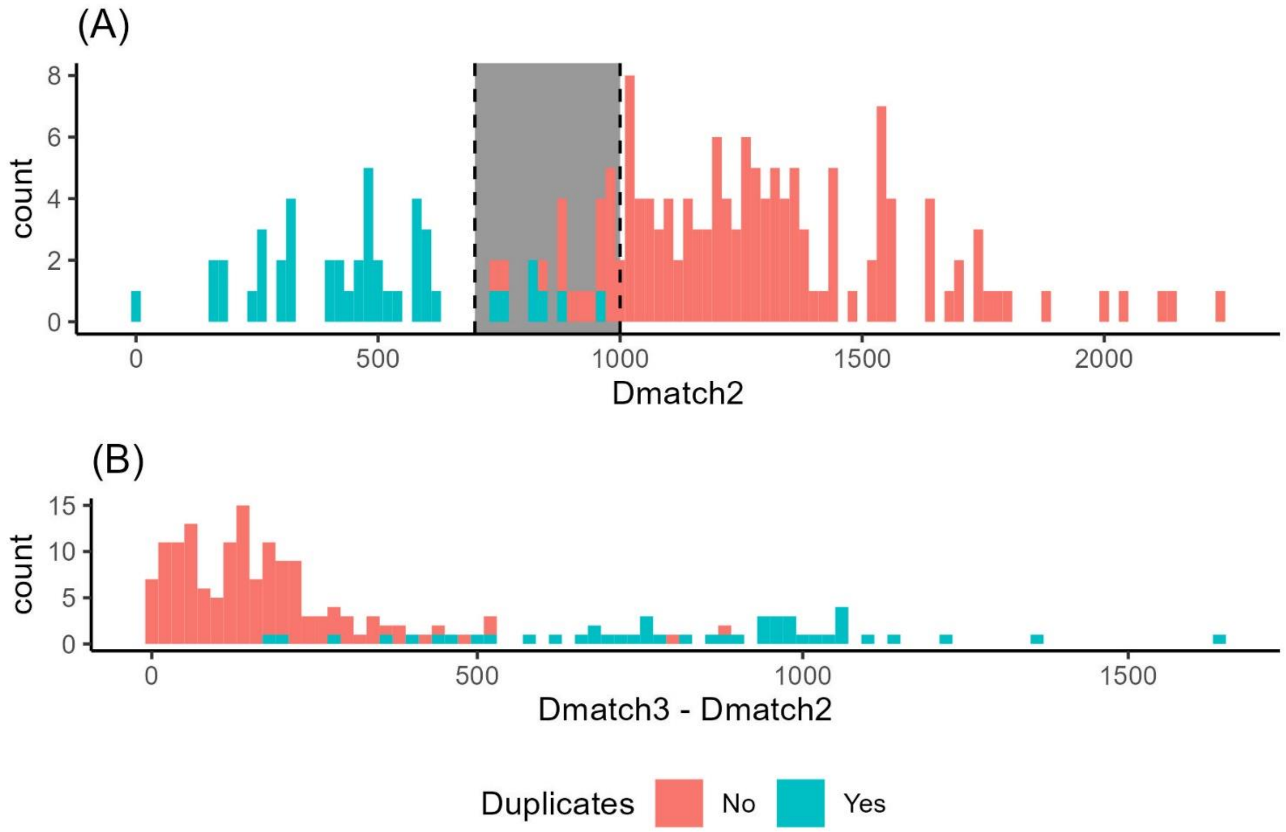

Figure S3. Distribution of scores in the field validation depending on whether the image is a duplicate (blue) or not (red). Note that Dmatch1 was always 0 (the same fingerprint), as the database was matched against itself. (A) Scores of the best match (Dmatch2). The gray zone corresponds to intermediate scores where Dmatch2 potentially overlaps between duplicates and non-duplicates. (B) Difference between the best match (Dmatch2) and the second-best match (Dmatch3).
