# Supplementary material for: Spotted! Computer-aided individual photo-identification allows for mark-recapture of invasive spotted lanternfly (Lycorma delicatula)
Source: Front Insect Sci. 2023 Feb 6;3:1112551. doi: 10.3389/finsc.2023.1112551 (PMC10926401; doi:10.3389/finsc.2023.1112551)
Supplement: Supplementary Data Sheet 2 — Sensitivity analysis [file DataSheet_2.pdf]

Supplementary Material 2 for manuscript: Spotted! Computer-aided individual photo-identification allows for mark-recapture of invasive spotted lanternfly (*Lycorma delicatula*)

N. Belouard[1,2]*, J.E. Behm[1]

[1]Integrative Ecology Lab, Center for Biodiversity, Department of Biology, Temple University, Philadelphia, Pennsylvania, USA
[2]ECOBIO (Ecosystèmes, Biodiversité, Evolution), Univ Rennes, CNRS, Rennes, France

*** Correspondence:**
Nadège Belouard
nadege.belouard@gmail.com

Sensitivity analysis

When analyzing images of spotted lanternfly, the score of the correct match (same individual) is not expected to vary with sample size, since this score is mostly related to digitization error, photo angle and environmental conditions. The score of the best-ranked incorrect match (most similar other individual) is expected to decrease with sample size, as the more individuals are compared, the more likely it is to find similar individuals. This means that this method could be less effective at finding recaptures for high sample sizes. However, our prediction is that the lowest score for an incorrect match should stabilize as sample sizes increase due to the important variability in spot patterns.

We tested the effect of sample size on scores by pooling all the different individuals available from this study (N = 309, lab validation and field validation pooled). We sampled 10 individuals from this pool and matched them against this same pool of 10. Dmatch1 was always 0 (the same image), and we extracted Dmatch2 (scores of the most similar different individual). We repeated this procedure 10 times, and then repeated the entire procedure for sample sizes of 20, 50, 100, 150, 200, 250, and once for the entire pool of 309.

Results seem conform to our prediction: as sample size increases, the average Dmatch2 initially decreases linearly, then seems to decrease more and more slowly and may stabilize around a score of 1000 (Figure S4). Only a fraction of individuals would require visual comparison when using 900 as the upper bound of the gray zone (upper threshold used in the manuscript; Figure S5), and this fraction does not seem to increase linearly (Figure S6), e.g., on average 0%, 14%, 22.5% and 30.1% of individuals for sample sizes of 10, 100, 200 and 309, considerably reducing user input time.

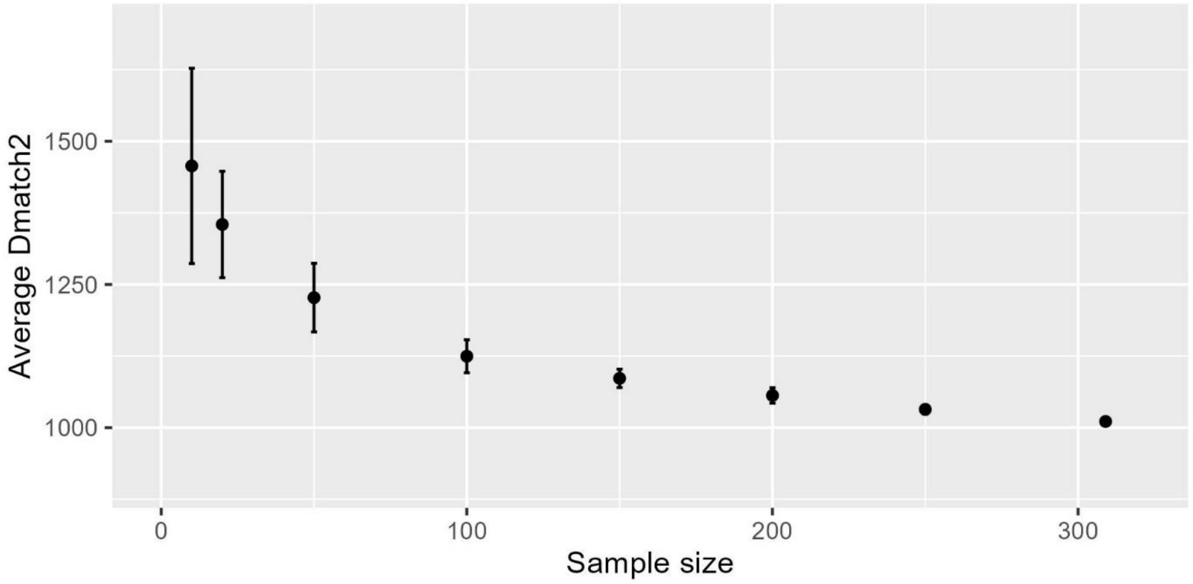

Figure S4. Evolution of the average Dmatch2 (score of the best-ranked different individual) with sample size when resampling the database of 309 individuals. Points and error bars represent the average ± SD values obtained from 10 replicates, except for the full sample size, N = 309 (no replicate).

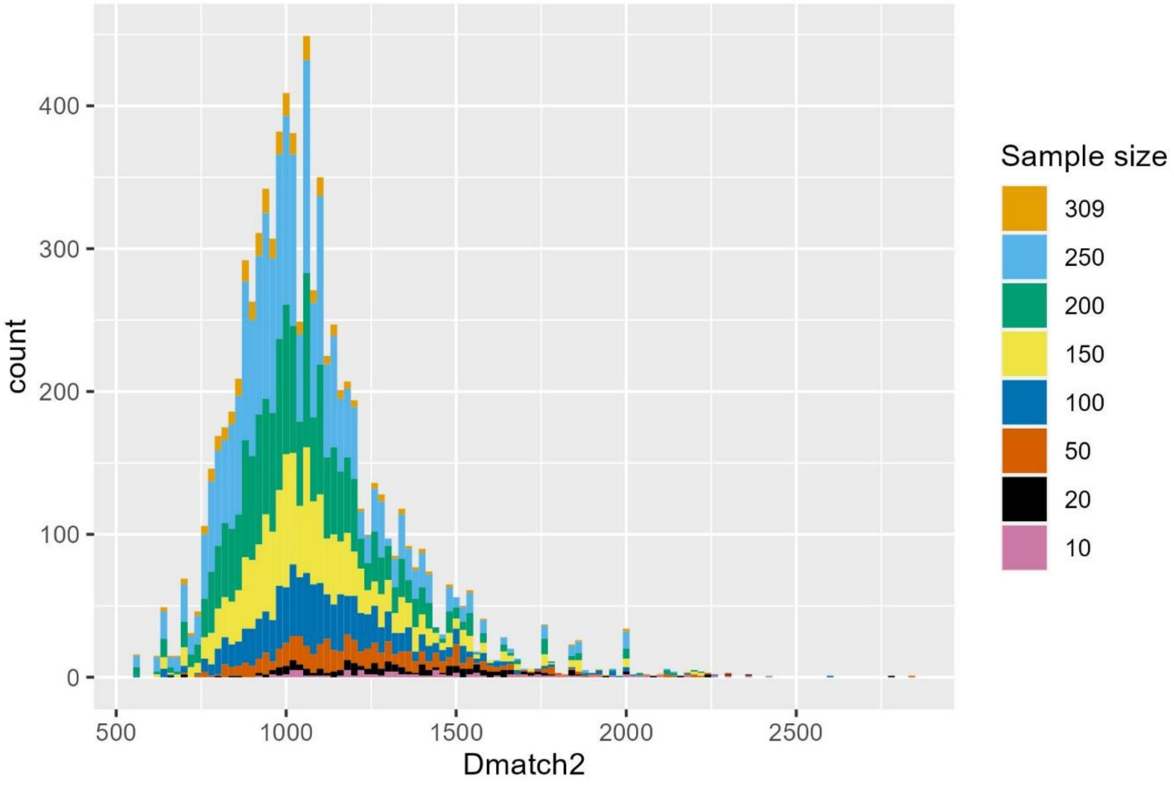

Figure S5. Evolution of the distribution of Dmatch2 (score the best-ranked different individual) with sample size when resampling the database of 309 individuals. Each distribution is the sum of all 10 replicates, except for the full sample size, N = 309 (no replicate).

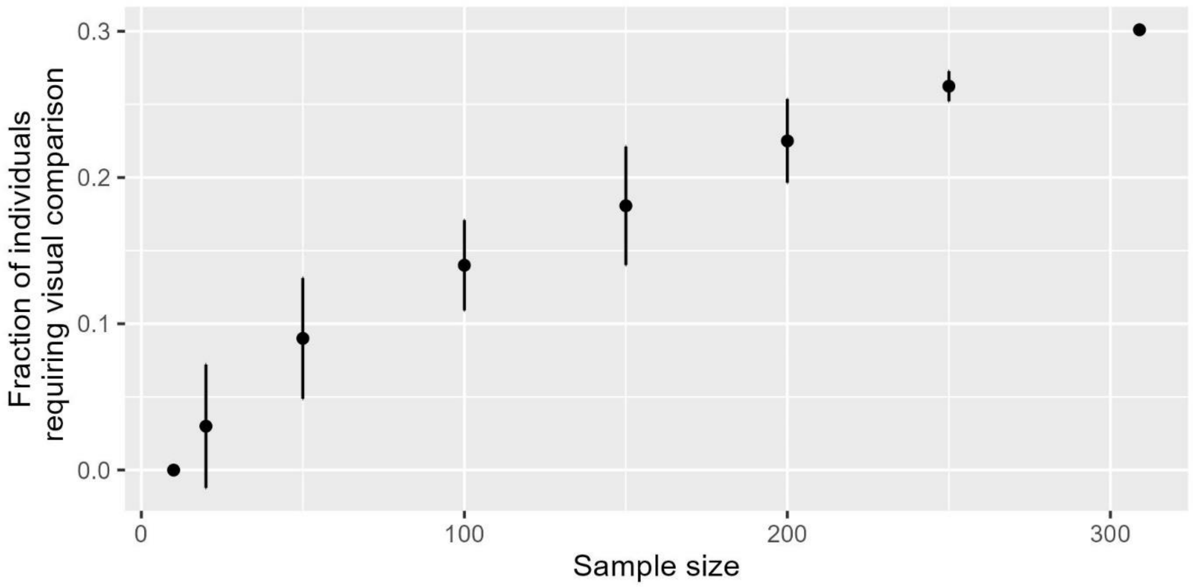

Figure S6. Evolution of the fraction of individuals with Dmatch2 < 900, requiring visual comparison, when resampling the database of 309 individuals. Points and error bars represent the average ± SD proportions obtained from 10 replicates, except for the full sample size, N = 309 (no replicate).
